# Bioengineered Skin Intended as In Vitro Model for Pharmacosmetics, Skin Disease Study and Environmental Skin Impact Analysis

**DOI:** 10.3390/biomedicines8110464

**Published:** 2020-10-31

**Authors:** Raquel Sanabria-de la Torre, Ana Fernández-González, María I. Quiñones-Vico, Trinidad Montero-Vilchez, Salvador Arias-Santiago

**Affiliations:** 1 Cell Production and Tissue Engineering Unit, Virgen de las Nieves University Hospital, 18014 Granada, Spain; raquelsanabriadlt@gmail.com (R.S.-d.l.T.); mariai.quinones@juntadeandalucia.es (M.I.Q.-V.); salvadorarias@ugr.es (S.A.-S.); 2 Biosanitary Institute of Granada (ibs.GRANADA), 18014 Granada, Spain; tmonterov@correo.ugr.es; 3 Andalusian Network of Design and Translation of Advanced Therapies, 41092 Sevilla, Spain; 4 Dermatology Department, Virgen de las Nieves University Hospital, 18014 Granada, Spain; 5 Dermatology Department, School of Medicine, Granada University, 18016 Granada, Spain

**Keywords:** animal experimentation, atopic dermatitis, bioengineered artificial skin substitutes, fibroblasts, product testing, psoriasis, regenerative medicine, tissue engineering, keratinocytes, transepidermal water loss

## Abstract

This review aims to be an update of Bioengineered Artificial Skin Substitutes (BASS) applications. At the first moment, they were created as an attempt to replace native skin grafts transplantation. Nowadays, these in vitro models have been increasing and widening their application areas, becoming important tools for research. This study is focus on the ability to design in vitro BASS which have been demonstrated to be appropriate to develop new products in the cosmetic and pharmacology industry. Allowing to go deeper into the skin disease research, and to analyze the effects provoked by environmental stressful agents. The importance of BASS to replace animal experimentation is also highlighted. Furthermore, the BASS validation parameters approved by the OECD (Organisation for Economic Co-operation and Development) are also analyzed. This report presents an overview of the skin models applicable to skin research along with their design methods. Finally, the potential and limitations of the currently available BASS to supply the demands for disease modeling and pharmaceutical screening are discussed.

## 1. Advances in Tissue Engineering

Nowadays, there is an enormous clinical demand of organ donors. The increasing of population life expentancy have caused a raise of the number of people who needs a transplant, limiting their availability. Only in the United States, eighteen patients die daily while they are waiting for an organ. This is related to the fact that every ten minutes one patient is added to the organ transplant waiting list, which only in this country exceed 113,000 people [1]. The way of deal with this constant demand of transplants needs to change radically.

In the last few years, the huge advances in differents sciences such as Biology, Medicine, Chemistry, Material Science and Engineering, has been opened a new way for the discipline called Tissue Engineering (TE). This name was officially coined by the National Science Fundation of Washington in 1987 [2].

The TE main target is the design of tissues and organs able to keep, to repare or to enhance the functions of their original ones.

As a sample of the great growth that this discipline has experimented recently and the resources that are being put into it, we do a search in PubMed with the keywords ‘’Tissue’’ and ‘’Engineering’’, and around 130,000 publications appear instantly. If we do the same in Clinical Trials, there are approximately 92 clinical trials.

One of the greatest challenges of this therapies is their clinical translation. It is necessary to take into account the legislation applied by the Food and Drug Administration (FDA), the orientation documents for the products created by TE and also the indications to follow in order to put into working the clinical trials. Moreover, the reglamentary requeriments for biological products used in TE follow the Good Tissue Practices (GTP) and Good Manufacturing Practices (GMP) regulations [3].

The new progress in the regenerative medicine area has been made with new laws, achieving the goal of obtaining more secure products, which leads to a significant improvement in these treatments. There are studies in orthobiologics, musculoskeletal defaults, urology and wound healing.

An encouraging example of this tissues and organs clinical translation is being carried out in Andalucia, where fourteen severely burned patients have received an autologous Bioengineered Artificial Skin Substitute (BASS) transplantation [4,5]. The results showed an 80% survivance rate and clinical, homeostatic and histological positive findings [6]. This skin model is manufactured in the Cell Production and Tissue Engineering Unit of Virgen de las Nieves University Hospital in Granada, and it shows the extraordinary potential of these advanced therapies, which are under constant improvement [6,7,8].

This review aims to collect the main applications of BASS for in vitro test, including pharmacosmetic development, skin disease models and environmental impact studies. The parameters frequently used to characterize these BASS are also reviewed.

## 2. Human Artificial Skin

The skin, the biggest organ in the human body, creates a barrier between external and internal environment. Apart from acting as a prime defensive line of the body against physical, chemical and biological agents, it also contributes to regulate the organism temperature, to keep the homeostasis, to participate in the mechanisms of sensorial perception, as well as in regenerative processes.

This organ is composed by three main layers, being the outer one, epidermis, mode out of five sublayers of cells called keratinocytes. These are produced in the inner basal layer and they move towards the surface experimenting a set of changes, called keratinization, until reaching the most external layer called stratum corneum (SC). This is waterproof and its main function is to avoid the entrance of pathogens inside the body. Next, the second layer is called dermis, formed by connective tissue whose main cells are the fibroblasts, which segregate collagen and elastin, adding strength and flexibility to the skin. Just below, the hypodermis, mainly composed of adipocytes, provides isolation and cushioning to the skeleton structures.

When the skin is damaged or harassed, all these functions are lost in the affected area. However, thanks to the presence of stem cells, the epidermis has the ability of self-regeneration to a certain extent. In the deepest wounds, such as second or third degree burns, the healing cannot be completed and chronic injuries are produced, leading to important disabilities or even causing death.

As a consecuence of the advances in TE, BASS are able to emulate the skin functions, and they can be classified attending to several criteria: cellular (autologous or allogenic origin) or non-cellular; epidermal, dermal or composed (dermoepidermal); natural (autologous, allogenic or xenogenic biomaterials) or synthetic (biodegradable or non-biodegradable) [9].

Among all the BASS currently available, cellular and natural substitutes have been proved to be the most effective. Furthermore, if the cellular origin is autologous, the feared immunological rejections are ruled out and infections are reduced. To build this type of tissue, it is important to have a deep understanding of cell culture and isolation.

Regarding the 2D skin model, epidermis and dermis, two cell types are mainly needed, keratinocytes for the epidermis and fibroblasts for the dermis. The connective layer is easier to produce, since fibroblast culture is simple, they grow rapidly in 2–3 days. In the case of the epidermis, keratinocytes have a low ratio of division in vitro. It is a challenge to obtain a method of keratinocytes isolation and culture that is also not contaminated by fibroblasts. In addition, the culture of keratinocytes can be contaminated by other cell types, such as Langerhans cells or melanocytes, but this contamination is virtually insignificant and these cell types are much easier to remove from the culture. In most cell isolation protocols, both layers are separated by physical and enzymatic treatments, the latter is usually carried out with trypsin [10]. Subsequently, a new two-step method was described in which the tissue was digested with a protease prior to treatment with trypsin [11]. This procedure was shown to decrease fibroblast contamination and improve the viability of keratinocytes (probably due to the shorter time in contact with trypsin) [12]. Even with all this, it has been demonstrated that, among all the keratinocytes that are isolated, only 3% that are under optimal conditions will form colonies [13].

Regarding keratinocytes cultivation, there is a gold standard method, described by Rheinwald and Green in 1975 [14]. A feeder layer of 3T3 murine fibroblasts (MFs) irradiated by gamma rays is used, which lose their proliferative qualities but maintain their metabolic activity, providing to keratinocytes growth factors, toxin elimination, among other benefits. The keratinocytes are seeded on the irradiated fibroblast layer and a culture medium, which containing fetal bovine serum (FBS) and supplements that increase proliferation such as epidermal growth factor, cholera toxin (can be replaced by isoproterenol), hydrocortisone, triiodothyronine and insulin, is added to them [15]. This method, although with good clinical results, has quite a few limitations, the use of components of animal origin such as MFs or the FBS, imply serious immunological and infection risks.

As an alternative to the use of MFs, in 1989 a method was described to used irradiated human fibroblasts (HFs) as a feeder layer for the culture of human keratinocytes [16]. To completely remove the xenobiotic components from the culture it would be essential to also substitute the FBS from the culture medium. In this way, a totally clean method of xenobiotics would not be obtained since FBS is used in the culture of HFs [17].

Serum-free culture medium has also been produced, allowing the expansion of keratinocytes without the need for a feeder layer. One of them is the MCDB153 medium (Sigma-Aldrich, St. Louis, MO, USA), described by Boyce and Ham in 1983 [18]. The strategy used was to decrease the calcium concentration to 0.1–0.03 mM, at which the keratinocytes maintained their phenotype and the contamination by fibroblasts was greatly reduced. A high-calcium concentration induces the formation of intermediate keratin filaments, favouring the keratinocytes differentiation and their stop proliferation. In addition, supplements such as epidermal growth factor, transferrin, monoethanolamine, phosphoethanolamine, hydrocortisone and insulin were added [19]. However, this method does not achieve the optimum performance obtained by the standard method if chemically undefined bovine pituitary extract is not added [20].

Likewise, substrates capable of replacing the feeder layer have also been found. Among these substrates are fibronectin, collagens, vitronectin, synthetic membranes, surfaces conditioned by different cell types and dermal substrates [21].

Recently, a new chemically defined, serum-free, no feeder layer method has been described [19]. In this, biologically relevant recombinant human laminins (LNs) are used as substrates, specifically, LN-511 and LN-421; and the serum-free medium KGM-CD (Lonza) was used as the culture medium. The keratinocytes reached a great confluence after one week. They presented a normal phenotype and were identified with the baseline markers keratin 5 and 14 (KRT5, KRT14), integrin A6 and B1 (ITGA6, ITGB1) and the differentiation markers KRT1, KRT10 and involucrin (IVL). Finally, the efficacy of the method was demonstrated by grafting it into an animal model [19].

Once the cells are obtained, there are several ways to build different BASS. To get an idea, the BASS generated in our GMP facility, to which it was referred previously, consists of two layers, epidermis and dermis. The first layer is an epithelium made up of keratinocytes; the second layer is a fibrin-hyaluronic acid matrix with fibroblasts immersed. The lower layer (dermis) is manufactured 24 h before. The skin substitute is maintained in culture for approximately 1–2 weeks, and after that, it undergoes a pressure dehydration process in which it acquires its final thickness when water is removed [4,22].

This is a successful example within the multitude of procedures described for manufacturing artificial human skin. In fact, with new advances in science and technology, 3D bioprinting has reached TE, and there is also the possibility of designing BASS with bioprinters. This method is capable of generating highly complex and precise structures, being able to reconstruct the SC, the epidermis and the papillary and reticular dermis, and it is even working on the development of vascular networks, sweat glands and hair follicles [23].

Recently, a transcriptomic and proteomic analysis has been published comparing a bioprinted three-dimensional BASS with native human skin. Results showed an enormous similarity in signaling pathways involved in skin development and physiology, such as extracellular matrix (ECM) structuring, collagen fiber arrangement and keratinization [24].

Increasingly, the BASS recapitulate the native human skin more similarly, in its deeper biology and in the way it is structured. This has led them to have applicability in various areas. In vitro human skin is a powerful tool which allows the screening of cosmetic and pharmaceutical products, and it is also useful to know the relevant physiological processes for the skin. It provides the possibility of studying the transdermal behavior of ingredients/medications for topical application [24].

## 3. Applications

The primordial utility of these skin models has been grafting. There are commercial artificial skin substitutes [25] and autologous BASS manufactured under GMP conditions [4,7] to treat patients with burns. Furthermore, these BASS are also being used to analyze and take measurements of the physiological features of human skin [25]. Currently, there are also BASS designed to model skin diseases, which gives researchers and doctors access to closely analyze the disease at various levels.

**Figure 1:**
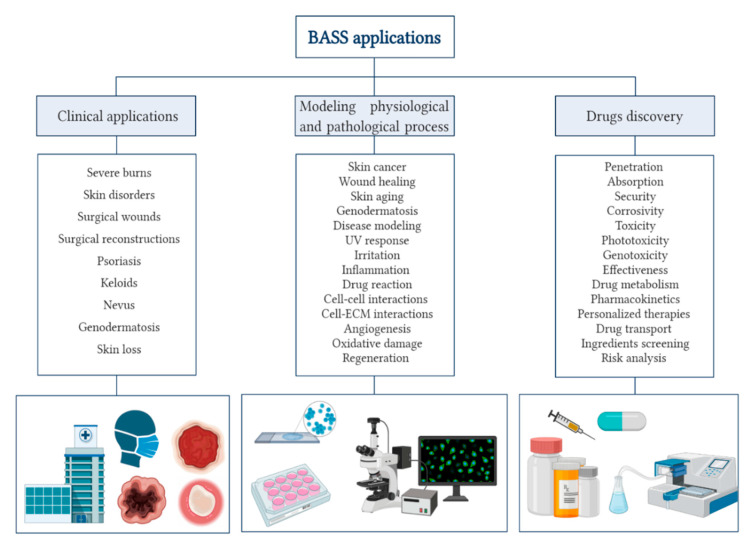
Diagram of the different applications of BASS: clinical applications, modeling physiological and pathological process and drugs discovery. Created with BioRender.com.

Figure 1 represents the different application areas where BASS models are appropriate and their specific trials in which they are effective.

### 3.1. Uses on Cosmetic and Pharmaceutic Research

Up to the date not all the desired tests can be combined with the use of artificial skin in vitro. This is an area that founds itself in great development. Although it is a fact that in vitro experiments are trusting models for simulate in vivo experiments, certain studies have pointed out significant inequalities in these methodologies to measure skin permeability against many compounds [26].

However, it is necessary to carry out a risk assessment before taking a compound for human use, test their toxicity, effectiveness, systemic exposure, etc, in order to have accurate knowledge and ensure consumer protection and safety.

The best rated and most reliable data in these studies are those carried out directly on humans in vivo, however, this is not sometimes possible, especially in the initial steps of obtaining a new pharmaceutical product. That is why for years these experiments have been put into practice on animals, and today, the development of in vitro skin models for this purpose is a great challenge.

The animal skin to take up in vitro experiments also shows serious disadvantages. Although it is true that pig ear skin is considered an appropriate model to make the attempt to permeability type tests, it is important to keep in mind that there are differences both in its mechanical properties (for example, tension) and in the way the environment affects (such as seasonal changes).

Another animal model used is the skin of the hairless rat [27] and the skin of the hairless mouse [28]. Analysis of percutaneous absorption of compounds on both showed that the in vitro skin of these animals provided measures not comparable with the data obtained in human skin in vivo [26]. Frequently, the data obtained from experiments carried out in animal models have limited validity. This is due to differences in metabolism as well as in the anatomy of the organism.

TE-designed BASS models have been developed to emulate fundamental structural and functional aspects of natural skin. The use of BASS models is an effective alternative to animal models. However, in vitro experiments on BASS monolayer models also have low relevance due to the lack of complex cell-cell and cell-ECM interactions [29]. But this limitation can be overcomed by the designing a three-dimensional physiological environment, with bilayer models, allowing the interaction of different cell types with each other and with the ECM.

Currently, skin substitutes are used in pharmacological research as reliable model systems to identify the irritating, toxic, or corrosive properties of chemical agents which come into contact with human skin [30].

Some companies are dedicated to evaluate the efficacy and safety of dermocosmetics with cellular and tissue models (ex vivo), which allow rapid and reliable predictive systems to be reproduced in the laboratory.

Both evaluations can be carried out to measure the mechanisms of action of an active ingredient, as well as studies based on conventional protocols following international guidelines (Table 1).

### 3.2. Alternative Method to Animal Experimentation

Over the years, the way of thinking towards animal experimentation has progressively changed [31]. A study conducted in 2014 stated that consumers, both in developed and non-developed countries, show concern for animal morality and welfare in the purchase of cosmetic articles [32].

To carry out a procedure, and to be sure that it can be performed repeatedly and in the long term, it is necessary to carry out an analysis of resources and possible deviations, as well as taking into account the ethics of the act performed [33].

In order to perform animal experimentation, it is extremely necessary to follow determined guidelines and give solid arguments to justify the experiment. It is also essential to determine the number of animals, the species, the study time, the methodology, the procedures to be carried out, … always ensuring the animal’s pain to be less or equal than the puncture of a needle.

According to the European Directive 86/609/EEC127 from 2003 and the 7th amendment of the Cosmetics Directive 76/768/EEC, animal testing for cosmetic ingredients has been forbidden since 2009, except for repeated doses toxicity tests, which were carried out until 2013 [34,35].

In this context, the famous 3Rs were introduced: Replace, Reduce and Refine. These principles establish that the tests must be carried out minimizing the stress caused to the animals, providing them with an adequate environment, medical attention and narcotics (Refine), the number of animals used in the experiments must be reduced (Reduce) and the analyses must be replaced by other in vitro methods (Replace) [36].

In contrast, substances increasingly require more tests to detect harmful side effects, as established by the new European Community regulation on chemicals and their safe use [36].

Furthermore, demand of the cosmetic industry is devastating, it is estimated that 5 billion of cosmetic products are sold annually in the United States by 2000 companies, and this study was conducted in 2009 [37]. Estimations made more recently (June 2019) by the Statista tool ‘Consumer Market Outlook—Cosmetics and Personal Care’, showed the average expense per person a year in different countries (Table 2).

However, five of the key areas in cosmetics testing (toxicokinetics, sensitization, repeated dose toxicity, carcinogenicity and reproductive toxicity) were absent from an alternative test to animal testing [38].

The decision established by the regulation about not to allow the use of experimental animals for this type of tests leaded to the urgent need to get other resources.

In this context, human skin and hair follicle ex vivo models are increasingly used in preclinical studies from various areas. For example, Bertolini et al. performed cytotoxicity and aging analyses in human skin ex vivo [39]; Gheraldini et al. validated caffeine as a treatment for hair follicles damaged by exposure to ultraviolet (UV) light in ex vivo skin models [40]. Fischer et al. carry out studies on the action of caffeine on hormonal pathways involved in the development of the hair follicle in skin ex vivo models [41]. It is demonstrated that are well predictive of in vivo effects [42].

Outside Europe, there are ethics committees that inspect animal testing, such as the Animal Ethics Committee (Canada), Institutional Animal Care and Use Committee (USA) and Ethics Committee (Australia) [43].

## 4. Human Artificial Skin: In Vitro Model to Research

BASS are used for different purposes that will be reviewed in this section. These models have been increasingly used in recent years both for testing products and for basic biological or pathophysiological research [44].

These BASS allow to investigate fundamental processes of the skin such as the epidermis formation [45], the interactions between cells, the healing process, stem cell maintenance [46] and pathogen infections [47].

As for applied research, the complex design of disease models has been a complete success, obtaining important studies for drug development and detection [48].

The BASS have almost completely replaced animal testing for cosmetic research. Most of these biological tests are regulated by the Organisation for Economic Co-operation and Development (OECD) (Table 3).

**Figure 2:**
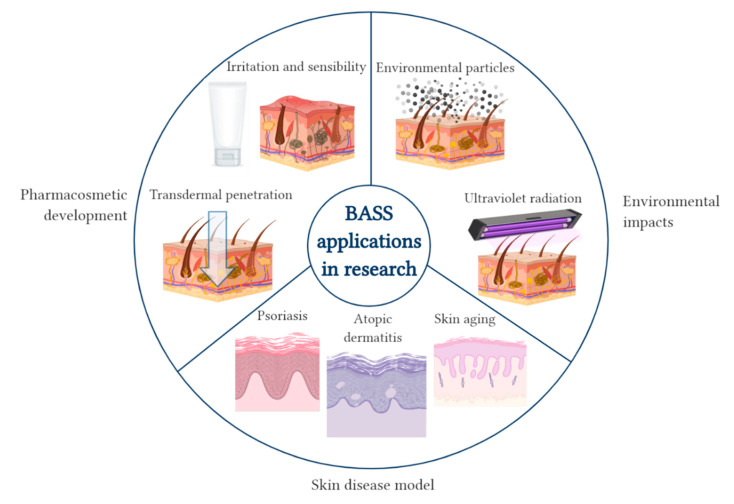
Diagram of the applications of BASS in research: pharmacosmetic development, environmental impacts and skin disease models. Created with BioRender.com.

In this specific section, BASS applications are explored in three research areas (Figure 2).

### 4.1. Pharmacosmetic Development

#### 4.1.1. Irritation and Sensibility Test

When a cosmetic is launched out into the market, it is essential to assess its irritation potential. The standard irritation test, both ocular and dermal, was established by Draize in 1944, and listed as Organisation for Economic Co-operation and Development Test Guideliness (OECD-TG 404 and TG 405) [49]. This consisted of administering the component to be analyzed to the eye or skin of a rabbit without anesthesia, and the subsequent evaluation of irritation by measuring redness, swelling, turbidity, edema, hemorrhage and discharge [50].

In order to maintain the precision of this test, but to avoid carrying out an invasive and violent process, several alternatives have been developed. Regarding the skin irritation test, cytotoxicity is evaluated in in vitro models, whether cellular or models of human artificial skin.

##### Cellular Model

The cellular model method consists of applying an established dosage of substance to be analyzed in a medium with human keratinocytes and quantifying the viability. The concentration of the substance capable of reducing the absorption of the neutral color (NR50) is the reference measure of the potential for irritation in the skin [51]. In this test, moreover, biomarkers can be used as references for irritation potential for interleukins-1 α keratinocytes (IL-1α) [52].

##### Reconstructed Human Epithelium

There are four models accepted by the OECD, which are EpiSkin™, EpiDerm™ SIT, SkinEthic™ RhE and LabCyte EPI-MODEL24 SIT. Kerastin™ is in a state of prevalidation [53].

In this type of tests, the chemical is applied in the model (made up of human primary keratinocytes). The damage caused by the agent will depend on factors such as the penetration of the agent through the SC, damage to the underlying keratinocytes, as well as the rest of the components of each model. The irritation potential is measured by signs such as the appearance of edema [47]. To assess cell viability, the MTT [3-(4,5-Dimethylthiazol-2-yl)-2,5-diphenyltetrazolium bromide, Thiazolyl blue; CAS number 298-93-1] is usually used, which measures the enzymatic reduction of tetrazolium to formazan in living cells [47]. In this context, it is better to use the AlamarBlue test, which is based on the metabolic activity of the cells and no formazan crystals are formed. Furthermore, it allows cell viability studies to be carried out over time since the reagent is harmless to cells. A cut-off level is established below which the chemical is considered to be irritating [53].

The lactate dehydrogenase (LDH) cytotoxicity assay is an effective method to be used in in vitro skin models. LDH is a cytosolic enzyme that is released after damage or lysis of cell membrane, by subjecting cells to a potentially toxic agent. The LDH released in culture media is measured and the percentage of cytotoxicity is calculated [54]. Recently, a modified LDH release assay has been optimized and validated to develop an in vitro human skin model useful for emulating a variety of skin-associated disorders [55].

##### Limitations and Perspectives

In vitro models for irritation tests are often delicate and easily damaged. Generally, these models feature human cells such as keratinocytes and fibroblasts, but do not use other cell types that may be essential in the process of in vivo inflammation [56]. Furthermore, influences at the systemic level, such as immunological or hormonal, cannot be addressed. Other limitations due to the use of in vitro assays may be the absence of innervation (no possibility of evaluating neurogenic inflammation), blood flow and immune cells (which are present in ex vivo models).

To overcome these obstacles, three-layered skin models have been developed, in which the skin barrier is improved and more appropriate in vitro test for irritants is designed. This system enables the topical application of substances, as well as the analysis of the influence on adipose tissue and the introduction of new endpoints like drug deposition would be possible [57].

Recently, Jusoh et al. propose an alternative skin irritation model based on a 3D method with a platform that allows the development of angiogenesis. For this, an irritating agent is applied in a model composed of keratinocytes, fibroblasts and endothelial cells immersed in microfluidic channels that allow paracrine and autocrine communication. The results showed that both sodium lauryl sulfate and steartrimonium chloride influenced angiogenic morphogenesis, improved the germination of blood vessels in the fibrin matrix and increased collagen deposition under inactive conditions. It is concluded that this platform represents a novel approach to replace current models of avascular skin irritation. In addition to irritants, this platform can be used to test other types of biochemical stimuli, such as allergens or corrosives [58].

#### 4.1.2. Transdermal Penetration Studies

A part of the risk assessment in the development of new products, either for cosmetics, medicines, or industrial chemicals, tries to analyze the dermal absorption potential, currently measured by in vitro techniques, using animal or human skin [59].

There are two types of techniques for evaluating penetration through the skin in vitro. On one hand, quantitative techniques include the use of diffusion cells and skin Parallel Artificial Membrane Permeability Assay (PAMPA). To carry out this type of evaluation, the BASS is placed between a donor compartment and another recipient. In the donor compartment, the solution to be studied is applied at fully controlled temperature and humidity, and then the solution reached by the receptor compartment is measured, that is, the amount of substance capable of crossing the skin barrier. On the other hand, qualitative or semiquantitative techniques are different microscopic and spectroscopic methods and their combinations. Their goal is usually to follow the active ingredient. The presence or relative amount of the active ingredient in the different layers of the skin can be determined. Using multiple methods together can seamlessly complement each other, facilitating the regulatory authorization process [60].

Transdermal penetration is highly influenced by the physiological and lipophilic characteristics of the compounds (when the lower is the molecular weight and the lower is the water solubility of the substance, the easier it is to penetrate the dermal layer). Penetration is enhanced when agents are diluted in water (25% for lipophilic agents and 10–20% for hydrophilic) [61]. Furthermore, it has been shown that tissue hydration increases the transdermal supply of substances [62].

A recent study by Zsikó et al. carried out a stability analysis of a lidocaine-loaded nanostructured lipid carrier (NLC) dispersion. They formulated an NLC gel and evaluated the lidocaine penetration profile through the skin. To study drug release and penetration, they used two types of Franz diffusion cells with three different membranes (a cellulose membrane, a Strat-M^®^ membrane and a heat-separated human epidermis). Furthermore, the NLC gels were evaluated using the Skin-PAMPA method, and the three methods were compared. Both the Skin-PAMPA model and Strat-M^®^ membranes correlated favorably with the heat-separated human epidermis in this research. However, the device, the membrane and the product properties, influence the penetration efficiency. The results pointed to NLC gel systems as the best in vitro test to model the penetration of human skin, but an adequate in vitro/in vivo correlation must be performed to calculate the drug release in vivo [63].

##### Penetration Studies through a Damaged Epithelium

The determination of dermal absorption in a damaged skin barrier is especially relevant in products such as sunscreens, creams for sun treatments, creams for irritated or diseased skin, such as those intended for atopic dermatitis (AD), which should only be applied to the skin that is sore or irritated [64]. It is likely that the damaged skin has a minor barrier function compared to healthy skin [65].

Tape stripping and cyanoacrylate stripping are methods used to sample SC in skin penetration studies [66,67]. In the tape stripping, a strip with the attachment is placed onto the skin and pressed using a rubber roller. The roller is moved 10 times back and forth, and then the tape is removed from the skin in one swift movement. The extraction of 10 subsequent strips is capable of modifying the properties of the barrier in the BASS causing an equivalent harm on the skin barrier in vivo [64]. This is the most appropriate method to make realistic predictions of the penetration of substance in the compromised skin [65].

In the cyanoacrylate stripping, glue is spread on the skin, an adhesive tape is placed on the glue under 10 N, and after 5 min, the tape is removed.

Dong et al. quantified the usefulness of both techniques to perform skin penetration studies. To do this, they used high-resolution multiphonic tomography, and determined that 50 tape stripping or 4 cyanoacrylate stripping eliminated all human SC. With the first technique, only the transition zone between the SC and the granular layer was reached, due to limited adhesion, while with the cyanoacrylate test viable skin layers were reached. It is recommended to use tape stripping a maximum of 30 times or cyanoacrylate stripping a maximum of 3 times to obtain skin with an ex vivo barrier that mimics diseased skin [67].

#### Limitations

The previous described methods are suitable for analyzing the penetration of lipophilic and hydrophilic substances. However, it must be taken into account that the finished products may present added substances (such as emollients), which increase the solubility or dispersion, affecting the penetration potential and therefore, the systemic exposure [64].

On the other hand, the amount of SC removed by tape stripping is influenced by the type of tape, the application pressure and the duration of the pressure. These influences prevent the comparison of the results obtained from different laboratories. The transepidermal water loss (TEWL) value is used to indicate the extent of the barrier interruption [67].

In this context, the diffusion of water through the skin can occur in both directions, by osmotic pressure. Therefore, there may be a flow of water from the donor compartment to the recipient. However, the influence of tissue hydration on skin permeability is more likely to be the main mechanism involved [56]. In this type of study, the use of human skin is essential, since its permeability when exposed to water differs from animal skin [64].

During the risk assessment process, from which the systemic exposure to substances is estimated, it is important to consider whether the product is going to be applied to damaged skin or not, since the systemic exposure may differ up to 10 times depending on said criterion, depending on the substance. Therefore, the BASS are useful for determining topical exposure, both under conditions in which the skin barrier is intact and when it is damaged [65].

### 4.2. Skin Characterization

Whatever the application to which a BASS designed by TE is going to be destined, it is essential to validate its integrity, assess its degree of similarity with human skin in vivo and its validity as a protective barrier.

To characterize BASS, three parameters indicated by the OECD have commonly been used; Tritiated Water Flux (TWF), TEWL and Electrical Resistance (ER). These three parameters are faithful markers of the cutaneous barrier function, all of them employing a Franz diffusion cell to carry out the measurement. They are used to validate the BASS and also to guarantee their effectivity.

#### 4.2.1. Tritiated Water Flux (TWF)

It has been the most used test years ago, however, it has been replaced by the other two methods.

This analysis involves applying an aliquot of a set volume of tritiated water (which has known radioactivity) to the skin surface in vitro. Upon contact of the liquid with the skin, time 0 is established, and measurements of the fluid are taken in the receiving chamber of the Franz cell, generally at times 3, 4, 5 and 6 h post-application. Once the measurements have been taken, the radioactivity of the fluid is analyzed.

The water flow is expressed as the coefficient of permeability Kp [68], and it is calculated:

$$Kp=\frac{\mathrm{water}\;\mathrm{flow}\;\mathrm{rate}}{\mathrm{applied}\;\mathrm{concentration}}$$

$$w\mathrm{ater}\;\mathrm{flow}\;\mathrm{rate}=\;\frac{\mathrm{disintegrations}\;\mathrm{per}\;\mathrm{minute}\;\left(\mathrm{dpm}\right)}{\frac{{\mathrm{cm}}^{2}}{h}}$$

$$\mathrm{applied}\;\mathrm{concentration}=\;\frac{\mathrm{dpm}}{\mathrm{mL}}$$

The TWF measurement has limitations; among them, water hydrates the SC, and in certain analyses, this is not convenient. In addition, these analyses are more time-consuming as appropriate regulatory approval is required to use radiolabeled substances [69]. For these reasons, in recent years, the measurement of ER and TEWL have replaced the determination of TWF in most laboratories [70,71].

#### 4.2.2. Electrical Resistance (ER)

Numerous studies point to it as the strongest and most rigorous test. The ability of the ions to flow through the skin is determined.

A voltage is applied to the skin for a few seconds through two stainless steel electrodes, one placed on the donor chamber of the Franz diffusion cell, and the other on the receiving chamber, thus establishing a circuit [70].

The ER of the skin is calculated using the voltage applied to the skin, the electric current and Ohm’s law. Electrical conductance is the reciprocal of resistance. The conductance value is usually normalized based on the surface of the skin [70]. The readings are expressed in kiloOhms.

$$\mathrm{Resistance}=\;\frac{\mathrm{voltage}}{\mathrm{electric}\;\mathrm{current}}$$

Davies et al. compared the traditional TWF method with ER. The study was carried out on 6 different species and with 2 types of skin preparation techniques. In practically all cases there was a good association between high TWF and low ER. This investigation strengthens the idea that ER is a fast and valuable tool for measuring the integrity of a variety of skin membranes without the need for radiolabeled material [72].

#### 4.2.3. TransEpidermal Water Loss (TEWL)

TEWL measures the amount of condensed water that diffuses through a fixed area of SC to the outside of the skin surface per unit time. A break in the skin barrier will result in an increase in TEWL, due to the lower resistance exerted by the damaged skin against water loss. The water that evaporates from the skin is measured using a probe that is placed in contact with the skin’s surface, which has sensors that detect the density of water vapor. TEWL can be measured using an open chamber, non-ventilated chamber, or condenser chamber [73]. Farahmand et al. point to devices that have a chamber without ventilation and devices with a condenser chamber as more effective methods than devices with an open chamber. This is because they are less affected by environmental influences which alter the flow of water vapor [74]. However, they all show a good correlation in the results of the TEWL measurements [75].

TEWL is a sensitive measurement affected by the properties of the surrounding microclimate such as ambient humidity, temperature or air flow, so it must be measured under controlled conditions [70]. TEWL varies significantly between anatomical sites, as it depends on aspects such as the sweat glands activity or the corneocytes properties. The SC contributes to the skin barrier properties and has a great impact on the protective function, exercising enormous control over the loss of transcutaneous water [76].

TEWL is the most widely used objective parameter to evaluate the skin barrier function in healthy individuals and also in patients with skin diseases associated with skin barrier dysfunction. Thus, skin diseases such as AD, psoriasis and thiosis are associated with increased TEWL. In fact, the measurement of TEWL during the first days of life is capable of predicting the development of AD during childhood. TEWL is therefore useful to identify infants with an increased risk of developing AD and thus establish preventive strategies such as the application of emollients [77].

TEWL has also been used as a quantitative parameter to assess the integrity and function of the skin barrier in the skin explant [72] and the formation of skin barriers in BASS [78]. Despite this, its effectiveness in determining the integrity of the skin in in vitro diffusion cells has been highly questioned [79].

However, it has been shown that the TEWL measurement can be correlated with the TWF and ER values [71]. TEWL and ER are appropriate parameters for detecting skin disturbance, both being cheaper and easier to use than TWF. The measurement of any of these three parameters allows quality control of the artificial skin samples.

Sometimes, it has been difficult to compare values measured in BASS with values taken directly in vivo, due to the great variety of factors that influence TEWL. In addition, each technique is adapted to carry out the measurements in a specific way. As a solution to this, numerous commercial companies have developed specialized probes to measure TEWL on in vitro plates, such as the case of Microcaya^®^. In this way, the data taken in BASS can be directly compared with the data obtained with the probes in the patients.

This way of standardizing the measurement is already carried out in the analyses. A recent example occurs in the percutaneous administration of medications. Colloidal particles were synthesized as a form of cutaneous drug administration and the variation of TEWL was analyzed both using BASS and in healthy volunteers. For in vitro measurements, the Tewameter^®^ TM 310 probe was used, which is perfectly adapted to carry out BASS measurements, since it emulates the donor compartment of Franz’s cell, leaning directly on the membrane as if it were resting on the skin. For the measurements taken in vivo, the probe used was Tewameter^®^ TM 300. The data obtained by both are comparable, providing precision and consistency to the analyses [80].

In conclusion, TEWL measurements in BASS is a simple and effective tool to carry out studies in dermo-cosmetics, in drug administration, in skin hydration analysis, etc. It is also used for tape stripping analyses, where TEWL values are increased by showing reduced structural integrity by removing corneocyte layers. In fact, with each consecutive peeling, a sharper increase in TEWL is observed. This demonstrates the sensitivity of the technique. It has also been used to view the kinetics of barrier recovery, evaluating the response to topical treatments and identifying metabolic processes that maintain a functional skin barrier [81]. And finally, since this parameter is fully established to detect skin diseases, TEWL measurements in BASS are useful to assess injury models of diseases such as AD or psoriasis. Thus, TEWL parameter assess whether BASS really emulate the properties of a skin in vivo with these characteristics, being useful to justify the validity of the in vitro injury models.

### 4.3. Skin Disease Models

Showing up next, Atopic Dermatitis, Psoriasis and skin aging models have been analyzed in terms of skin substitutes therapies.

#### 4.3.1. Skin Substitute with Atopic Dermatitis (AD)

AD is a chronic inflammatory disease of the skin that produces erythematous cutaneous lesions, as a result of the serious injury which severely impacts on the quality of life in affected patients [82]. It is related to genetic, immunological and environmental factors [83]. Its prevalence has been increasing over the past few decades, especially in developed countries, with a prevalence of 30% in child population and 10% in adults [84,85].

There are numerous treatments, even though there is a lack of adherence to them [86]. A novel method used today to search for new therapies for AD consists of using human epidermis reconstructed in vitro.

To design it, primary human keratinocytes are extracted. These are isolated from the epidermis, grown, expanded and then mature in an air-liquid interface, giving rise to a histologically stratified human epidermis and functionally equivalent to native human epidermis [87]. To induce AD in the epidermis, it is sufficient to modify the components of the culture medium [83].

The keratinocytes of the epidermis synthesize proteins such as IVL, loricrin (LOR) and filaggrin (FLG) [88], as they are all involved in the development of the SC of the afore mentioned layer. Low levels in the concentration of these proteins are combined with damages on the skin barrier that leads to the AD [89]. Mutations have been observed in the FLG gen in more than 20% of patients with AD [90].

Furthermore, patients with AD present a reduction in long chains, an increase in unsaturated free fatty acids and a decrease in long chains of ceramidas in the skin [91]. Inflammation produced in AD is correlated with overexpression of Th2 cytokines (IL-4, IL-5 and IL-13), IL-22 or thymic stromal lymphopoeitin (TSLP) [92].

Due to the multiple factors which can generate AD, a reconstructed human epidermis that emulates all the components of the disease cannot be developed, but is usually used to study specific aspects of it [83]. Numerous groups have developed their own AD models, each one modifying the culture medium in a different way to induce the disease. Despite this, no model reproduces all the characteristics of the AD. Since each model focuses on one or a few features of AD pathophysiology, it is important to choose the correct model according to the aims of the analysis [83].

One of the main disadvantages of these models is the absence of fibroblasts, immune cells and nerve endings. To mimic inflammation, cytokines are often added to the culture medium, which has so far been successful [83]. The reconstructed human epidermis has provided numerous advantages in the study of diseases such as AD, allowing the evaluation of the treatments developed by the pharmaceutical industries with high performance, while avoiding the use of animals [83]. Skin explant models allow keratinocytes to contact their environment (mainly fibroblasts), making them more objective [93].

In a recent study, the effect of dipotassium glycyrrhizinate (KG), an anti-inflammatory agent widely used for the treatment of AD, was analyzed. To do this, they induced AD in 2D BASS models (with epidermis and dermis) using Th2, IL-4 and IL-13 cytokines. KG decreased AD-related gene expression in Th2 cytokine-stimulated keratinocytes. KG alleviated AD phenotypes and gene expression patterns and inhibited AD-related cytokine release in disease models [94].

Liu et al. developed by 3D bioprinting a BASS model with vascularized AD. This BASS contains human keratinocytes, fibroblasts, pericytes and endothelial cells derived from induced pluripotent stem cells. Thanks to the increased cellular and physiological complexity, clinical characteristics of the disease were obtained, such as: (i) spongiosis and hyperplasia; (ii) early and terminal expression of differentiation proteins; and (iii) increases in proinflammatory cytokine levels. The effect of the possible pharmacological treatments for AD was tested in the model, and the results obtained in said BASS were correlated with clinical therapeutic data [95].

#### 4.3.2. Human Psoriatic Skin Substitute

Psoriasis is a chronic skin disease represented by red squamous plaques that usually appear on the elbows, knees, sacroiliac region, nails and scalp [96]. In addition to the evident pain and itching caused by plaques, there is an extensive list of comorbidities associated with this disease (including Crohn’s disease, psoriatic arthritis, atherogenic dyslipidemia, hypertension, diabetes, as well as increase of the carotid intima-media thickness (IMT)) [97,98].

About 7.4 million people in the United States and 125 million people worldwide are affected by psoriasis [99], being considered one of the most common skin diseases.

It is fundamentally associated with immunological and genetic factors, which makes it a heterogeneous disease without cure. Treatments are diverse and tend to be aimed at reducing skin lesions, being currently a source of research [100].

In this sense, in vitro models seem to be effective tools to investigate specific metabolic and physiological pathways [101]. Reconstituted skin substitutes in monolayer (epidermis) or bilayer (dermis and epidermis) are often used [102]. The bilayer substitute can be manufactured with keratinocytes and fibroblasts derived from psoriatic patients, either derived from healthy tissue or obtained directly from psoriatic plaques, giving rise to non-lesional and lesional psoriatic models, respectively.

To validate these models, panels of genes and proteins associated with the disease have been carried out. In this way, a list of the genes is drawn up whose regulation is most affected by the disease, which influence the induction and/or progression of psoriasis [103]. In addition, a relationship can also be established between the mentioned genes and the altered biological processes.

Misregulated genes have been found in various investigations among reconstructed skin substitutes with cells from a healthy individual, compared to an individual with psoriasis [103]. However, the number of deregulated genes is lower if the reconstructed skin substitutes from healthy patients are compared with the non-lesional psoriatic model, in contrast to when these substitutes are compared with the lesional psoriatic model. Still, the healthy skin of a healthy individual has a different pattern of genetic expression than the healthy skin of a psoriasis patient [104].

Specifically, Rioux et al. found 3540 genes with different genetic expression between healthy substitutes and psoriatic skin substitutes. Among them, 2850 genes were deregulated with respect to the lesional substitute, and 690 genes were deregulated against both psoriatic skin substitutes [102].

Differences in cytokines expression and growth factors were observed between the substitutes in the analyses, but these lacked for immune cells, and the cytokines were therefore expressed by keratinocytes [105].

Likewise, the genes related to LOR, KRT1, KRT2, KRT5, KRT31, KRT77, IL1A and IL1B were highly deregulated in the psoriatic skin model compared to those of the non-lesional models [106,107].

Of all the deregulated genes, most were related to the keratinization process, followed by isoprenoid metabolism and finally by retinoid metabolism. Regarding the deregulated genes between the lesional and non-lesional substitutes, they were mainly associated with the formation and development of the epidermis [102].

The cells that produce neuropeptides and hormones respond to neurotransmitters to contribute to the skin and the general body homeostasis maintenance. In the reconstructed models of skin these neuroimmunoendocrine connections are absent. Nevertheless, keratinocytes, melanocytes and cultured fibroblasts have been proved to be capable of producing corticotropin releasing factor, pro-opiomelanocortin and their corresponding receptors [108]. In addition, the production of cortisol and corticosterone in keratinocytes, melanocytes and cultured fibroblasts has also been demonstrated [109]. Therefore, in vitro models, both in monolayer and bilayer, can be useful for this type of study, as it is important to analyze interactions at all levels.

Recently, Simard et al. designed psoriatic BASS models and determined that the strongest phenotype of psoriasis was generated when cAMP levels were high, which occurred when cholera toxin was added as an additive to promote the proliferation of keratinocytes [110].

These substitutes have allowed us to go deeper into illnesses like psoriasis, despite the fact that these are incomplete models with lack of vascularization, innervation and immune cells [111]. On the other hand, there are substitutes which are manufactured only with keratinocytes and fibroblasts, since the analysis of the profile of genetic expression is reliable, because there are no other cell types in the model [102]. These models are a great advance in the development of new treatments for psoriasis. In fact, brazilin, an active compound of *Caesalpinia sappan* L., is capable of reinforcing the skin barrier in psoriatic models induced by TNF-α thanks to its anti-inflammatory activity [112].

#### 4.3.3. Skin Aging Model

Several mechanisms are involved in aging. These include the accumulation of mutations in genetic material, the accumulation of toxic metabolites, the establishment of free radicals causing oxidative damage, chemical modifications and the crosslinking of macromolecules by glycation [113].

BASS are exemplary models for aging studies since the skin is an organ exposed to both extrinsic influences, such as UV light, and intrinsic influences, for example of genetic origin [114].

When aging of the skin occurs, there are visible histological changes such as the reduction of the thickness of the epidermis, changes in the composition of the dermis, disorganization in the components of the ECM, the dermoepidermal junction becomes flat, etc.

There are different ways to approach the development of skin aging models. The most common forms are either to produce photoaging by exposure to UV light, or to generate chronological aging through glycation or modification in the collagen composition of the dermis. In the first, collagen is pre-exposed to UV radiations for the induction of dermal modifications. In the second, collagen is pre-incubated with sugar (ribose or glucose) prior use for preparing reconstructed skin [115].

New methods for creating BASS models of aging are produced considering the different populations of dermal fibroblasts and their changes during aging. Specifically, populations of papillary fibroblasts seem to disappear or at least decrease during aging, while reticular fibroblasts are not altered [116]. In this way, a reconstructed skin model with a dermis that only has reticular fibroblasts is a reasonable approach for studies of skin aging [117].

It is possible to combine factors to create more realistic models of skin aging. For this, BASS models can be designed with the glycosylated dermis and exposed to UV radiation [118].

Attempts have also been made to create aging models incorporating human skin cells corresponding to genetic diseases such as Werner’s syndrome [106]. Indeed, it is of great interest to create in vitro BASS models of genetic diseases by adding isolated and amplified cells from patient biopsies [119].

Thanks to skin aging studies, it has been shown that aging depends on factors such as age, ethnic origin, calcium content, sun exposure, etc. It has also been seen that by modifying the components of the ECM, the phenotype of fibroblasts can be changed. This is interesting to try to design a rejuvenation model of dermal fibroblast populations by modifying ECM [115].

The great advantage of these models is the contribution of new knowledge in the events occuring during aging. The downside is that aging is a long-term phenomenon while in vitro cultures are limited in time.

These models have an important applicability in cosmetics. They have been used to discover new candidate ingredients with anti-glycation action [120].

Furthermore, thanks to aging BASS models, some anti-aging molecules have been characterized, such as the substance P-based hydrogel. Exactly, it was demonstrated that treatment with this gel increased the levels of type I procollagen, correlated with the regulation of matrix metalloproteinase-1 (MMP-1) and tissue inhibitor metallopeptidase 1 (TIMP-1), as well as with anti-inflammatory effects. The effect of the gel is clear in wound healing, as it induces the regeneration of skin cells and the synthesis of collagen. Consequently, a new cosmetic ingredient with anti-aging action is discovered [121].

Theophylline, a drug used to treat asthma, has also been evaluated thanks to ex vivo skin models. By adding this compound to the human skin culture medium, there was a decrease in the apoptosis levels of epidermal keratinocytes, an increase in melatonin, a potent ROS protector, and an increase in KRT15, marker for basal keratinocyte. Theophylline, therefore, has important antioxidant properties, and could be a good candidate for skin anti-aging [39].

In a recent study, the use on a massive scale of a bilaminar BASS model, T-Skin™, is proposed as a system for the development of new cosmetics and dermatologically active ingredients. In this study, Bataillon et al. demonstrate that T-Skin™ is capable of reproducing a well-differentiated and organized epidermis and a functional dermis. The known favorable effects of retinol and vitamin C in this model had results that correlate with its anti-aging skin properties in vivo, showing the predictive capacity of the model [122]. Research on aging markers using T-Skin™ and subsequent human clinical evaluation have already been used successfully for the development of a new cosmetic active ingredient against aging [123].

### 4.4. Assessment of Environmental Impacts on the Skin

#### 4.4.1. Impact of Fine Environmental Particles

Fine particles are a complex and heterogeneous mixture of substances that pollute the air and put human health at risk. Most studies are focus on its impact on respiratory and cardiovascular diseases. Regarding skin diseases, fine particles are capable of promoting and even worsening conditions such as AD, acne, psoriasis, premature aging of the skin [124]. These particles are englobed by the term of exposome which is refered as the sum of external factors to which an individual is exposed throughout its lifetime. These external factors include, in addition to fine particles polluting the air, UV radiation, allergens, drugs or humidity among others [125,126,127]. In acne, exposome factors impact on severity and treatment efficacy. For instance, the harmful effect of air pollutants is more marked in acne patients [125]. Skin aging exposome include sun radiation, air pollution, tobacco smoke among others [126,127]. Skin care routines and sunscreen use may help to prevent this process [127]. Air pollution due to traffic has been associated with the development of eczema in children and facial lentigines, as well as with the modification of the integrity of the skin [128]. In fact, AD prevalence is increasing in areas of urbanization and cross-sectional studies have confirmed an association with higher levels of traffic-related air pollution and AD prevalence in both urban and small-town settings [129]. These air pollutants exert a harmful effect on the skin by increasing oxidative stress inducing severe alterations of the normal functions of lipids, deoxyribonucleic acid and proteins in the human skin [125]. At the cellular level, damage occurs to the dermoepidermal junction as well as a reduction in the vascularization and innervation of the dermis [130]. Between these particles travel nitrogen oxides, hydrocarbons, fine particles and ozone, capable of activating oxidative stress that induces damage to the skin, especially in sensitized skin [131]. Animal studies have commonly been used to study mechanisms such as the generation of reactive oxygen species (ROS), the positive regulation of aryl hydrocarbon receptor (AhR) profinflammatory factors and the positive regulation of keratinocytic vascular endothelial growth factor (VEGF) [132]. Identifying an environmental factor common and contributory to a population affected by a disease might enable large-scale preventative approaches.

More and more research is opting for models of BASS. For example, Pan et al. [133] looked at the effect of fine environmental particles (rich in inorganic metal elements, correlated with intracellular production of ROS), on skin tissue. For this, they used a monolayer model of sensitive skin in vitro; it only had epidermis. Specifically, keratinocytes were added in polycarbonate filter inserts in a serum-free medium. This model was also used to analyze the irritating and/or corrosive properties of some chemical compounds [134].

This study made it possible to determine that the fine environmental particles are not capable of inducing changes in the structure of the skin, but they are capable of causing a decrease in cellular functionality. The particles led to the production and release of IL-1α and IL-8, causing tissue inflammation and increased oxidative stress. Elevated levels of MMP-1 and MMP-3 were also detected, suggesting that the ECM was undergoing increased degradation. Exposure to environmental fine particles also caused reduced levels of gene expression related to cell junctions, anchors and terminal differentiation. Expressions of KRT1 and KRT10 decreased, so a skin barrier dysfunction was deduced [133]. Likewise, genes linked to apoptosis were also analyzed. BIRC5 (Baculoviral IAP Repeat Containing 5), the gene responsible for regulating the cell cycle and blocking apoptosis, was negatively regulated. CASP3 (Caspase-3) and CDKN2 A (cyclin-dependent kinase inhibitor 2A), pro-apoptotic genes, were upregulated. Cell death was the main cause of skin alteration [135].

BASS model was useful to study the premature aging of the skin as well as the loss of the protective role of the barrier. The main limitation of the model was its rapid aging, which meant that the exposure to pollutants was not long.

These types of studies show the usefulness of in vitro artificial human skin models to assess the chemical components of the environment, such as air pollutants and even tobacco smoke, and their influence on this organ. Furthermore, this model allows to take into account all levels (tissue, cell, molecular) to better understand the underlying mechanisms [135].

Experimental studies with BASS models have shown that phenolic extracts derived from plants have antioxidant and anti-inflammatory effects on cells exposed to airborne particles. Plant phenolic compounds decrease ROS levels in cells and/or improve cellular antioxidant capacity and, therefore, antennuate oxidative damage in nucleic acids, lipids and proteins [136].

#### 4.4.2. Impact of Ultraviolet Radiation

UV radiation is a potentially toxic environmental agent, with known harmful effects on human health, including skin diseases, immunosuppression, photoaging, cataracts and skin cancer [137]. In AD, a positive correlation between country-level monthly minimum and mean UVR exposure and AD prevalence was seen among 13- to 14-year-olds [128] and in acne, intensive UVR may trigger inflammatory acne flare-up [125].

UV is classified based on its wavelength in UVC (200–280 nm), UVB (280–320 nm) and UVA (320–400 nm). The UVC spectrum is fully absorbed in the stratospheric ozone layer. UVA and UVB reach the Earth’s surface. UVA represents 95% of the UV spectrum that reaches the Earth, and UVB represents 5%. However, UVB energy is absorbed more efficiently by cells, causing damage at significantly lower doses [138]. A 50% of UVA and a 14% of UVB rays are able to reach the skin layer where the melanocytes are located and the dermis [139].

Due to prolonged exposure to UV from the sun, the incidence of skin cancer has increased exponentially in recent decades. This radiation is closely related to the onset and progression of photocarcinogenesis, in fact, it is the main factor causing melanoma. Exposure to UV radiation gives rise to molecular and cellular mechanisms that generate DNA damage, epigenetic modifications, oxidative stress and inflammation [140].

In vitro investigations are carried out to study the cellular and molecular circuits related to photocarcinogenesis. The use of cell cultures allows to control the study environment. Cellular models with keratinocytes, melanocytes and fibroblasts stand out, used for the analysis of modifications induced by UV radiation in gene expression, shortening of telomeres and signal transduction [140].

This type of model has been used in numerous investigations. Thanks to them, UV has been correlated with keratinocyte apoptosis, the transport of nucleoprotein autoantigens to the cell surface of keratinocytes and the release of inflammatory cytokines like tumor necrosis factor α (TNF-α), IL-1, IL-6, IL-8, IL-10 and IL-17 [141]. Numerous pathways have also been characterized, for example Sestrin 2 has recently been discovered to be a positively regulated protein in human melanomas via the p53 and AKT3 (AKT serine/threonine kinase 3) pathways [142].

Similarly, the process of DNA breakdown due to UVC exposure [143], the role of hyaluronan synthase-2 against apoptosis caused by UV irradiation [144], and models of photoaging by irradiating BASS in vitro with different doses of UVA rays [145], have been put under research.

Reconstructed human epidermis models are also a great ally to determine compounds and ingredients that decrease skin aging. These are recommended for photoprotection studies [146]. One of these models allowed the demonstration that coumestrol has a preventive effect on photoaging of the skin [147]. Other BASS models have underlined the protective effect of bilberry extract against UV light, discouraging DNA fragmentation and inhibiting the expression of inflammatory factors [148]. Additionally, a natural killer cell conditioned medium (NK-CdM) has been found to possess potent antioxidant activity on the skin by inhibiting collagen degradation. It has also been shown to have anti-wrinkle effects by inhibiting UVB-induced MMP-1 expression. Therefore, NK-CdM is an effective therapeutic candidate to prevent photoaging of the skin [149].

Goyer et al. designed a pigmented BASS model by adding melanocytes at different concentrations. The amount of melanin in the BASS was inversely correlated with UVR-induced cyclobutane-pyrimidine dimer formation in dermal fibroblasts and keratinocytes. Pigmentation conferred by melanocytes protects against UVR-induced DNA damage [150].

These models are excellent systems for characterizing the effects of UV on the skin, including the study of carcinogenesis. A new in vitro model has even been described to investigate early melanoma invasion, as occurs in melanoma in the phase of radial and vertical growth [151].

## 5. Perspectives

Until now, the preclinical development of topical cosmetics and drugs has been supported by animal experimentation. Advances in TE contributed to obtaining different types of BASS, which have been developed with the aim of providing a solution to the treatment of large burns and chronic wounds. These substitutes made their way in a totally different investigation, but equally relevant [152]. On one hand, these BASS entered into toxicity analysis laboratories and have replaced all the experiments for the cosmetics discovery and a large part of the studies for the new drugs development. Furthermore, disease models have been designed, such as the examples described in this review. It should be noted that diseases not related to the skin, but to neighboring mucosal tissues, have even been studied [153].

BASS models have been shown to have numerous advantages over in vivo models, since faster, easier and more reproducible methods are carried out. This has led to their rapid incorporation into compound screening at high performance. It is essential to highlight that the results so far have demonstrated the predictive validity of the pharmacological and toxicological effects on the skin [44].

An important advantage of BASS is that the cellular composition is completely controllable by the researcher. Therefore, a certain cell type can be integrated or omitted depending on the purpose of the investigation. This is useful in determining the importance of cell type in a specific biological process. For example, to test possible immune reactions in the skin, Langerhans cells can be introduced into the model [154]. In addition, BASS allows the histological analysis of the sections of the skin models in vitro, which provides numerous data.

The possibility of manufacturing different BASS with their own characteristics of gender, age, ethnicity, anatomical site, etc. is interesting. Features that differ between groups, in order to study specific compounds or treatments directed to these specific groups, are within the area of we know today as personalized medicine. For example, the skin of white women has a greater tendency to develop wrinkles in the post-menopausal period, while black women have a greater stiffness in this epithelial tissue [155]; another example is pruritus, which affects black skin more than white skin [156].

In fact, Girardeau-Hubert et al. underscore the importance of taking the origin of cells in the development of BASS models into account. Caucasian keratinocytes underwent improved terminal differentiation in vitro compared to African keratinocytes, as evidenced by histological, transcriptomic and proteomic results. This fact may be related to the lower level of filaggrin detected in the African epidermis compared to the Caucasian epidermis. The differences in the in vitro behavior of keratinocytes according to cell origin suggested that they could contribute in part to the specific physiology between skin types and could be crucial as a strategy to study and adapt cosmetic products to different skin types [157].

Currently, there are still some challenges to be solved in the development of skin substitutes. A great challenge for researchers is the enormous complexity of this organ, aspects such as vascularization, sensory reception are still lacking and aspects of ECM must also be improved. The bio-printing technique seems to guarantee that the vascularization aspect will soon be advanced. In addition, to improve sensitivity, materials such as graphene, carbon nanotubes or nanowires are tested. Recently, the construction of 3D nerve networks in vitro with neurons derived from induced pluripotent stem cells (iPSCs) has been proposed. These iPSCs are generated from human skin fibroblasts. In this way, an innervated in vitro BASS is obtained, a characteristic that makes it at the forefront in modeling skin diseases while using cells derived from patients [158].

Skin research area still has a long way to go, and BASS seems to be the key tool for its advancement. The development of artificial skin has undergone a great evolution since its introduction in the 1980s. BASS allows researchers to obtain valid information on fundamental aspects of this important organ, while reducing animal experimentation and bring us closer to compliance with current regulations.

## Figures and Tables

**Table 1:** Pharmacovigilance test in pharmacocosmetics ^1^.

Toxicity Test	Genotoxicity Test	Efficacy Trials	Other Tests
Eye irritation (Invittox hemolysis test n 37, Model test of reconstituted human skin, Fluorescein leakage test method) Dermal irritation OECD 439 Dermal corrosion OECD 431; OECD 435 Nasal irritation Oral irritation Phototoxicity OECD 432 Cytotoxicity (MTT, XTT, Neutral red, Alamar Blue, LDH, MTS, Wst-8)	Ames test OECD 471 Mouse lymphoma assay OECD 476 Micronucleus test OECD 487 Comet assay	Cleaning efficacy Anti-angiogenic efficacy Anti-inflammatory efficacy Antioxidant efficacy Cell proliferation assay Cell cohesion test Anti-itch efficacy Lipolytic efficacy Collagen synthesis enhancement test Elastin synthesis test β-defensin synthesis increase assay Epidermal protease release assay Metalloprotease decrease assay Anti-growth factor action test Test of decrease in levels of human cathelicidin Healing efficacy Thermo-protective efficacy Reparative and regenerative efficacy Efficacy of anti-protective against dryness Moisturizing efficacy Antimicrobial efficacy	Percutaneous absorption and penetration OECD 428 Challenge test Microbiological controls Determination of active ingredients by chromatography Development and validation of analytical methods Nanotoxicology

^1^ Tests carried out to evaluate efficacy and toxicity of drugs and cosmetics: toxicity test, genotoxicity test, irritation test and other tests carried out.

**Table 2:** Cosmetics spending ^2^.

Country	Average Expense
China	32.14
Saudi Arabia	109.37
Spain	151.93
Italy	160.84
Germany	182.19
United Kingdom	206.06
United States	223.05
Switzerland	246.52
Japan	260.62

^2^ Average economic cost per person and year in cosmetic products.

**Table 3:** In vitro tests regulated by OECD ^3^.

OECD n	Trial Name and Description	Cell or Tissue
428	Skin absorption	Human or rodent skin
430	Dermal corrosion	Rat skin discs
431	Skin corrosion	Untransformed human keratinocytes
432	Phototoxicity	3T3 cells
435	Dermal corrosion	Aqueous protein gel membrane
439	Skin irritation	Epidermal keratinocytes
442D	Dermal sensitization	Immortalized transfected stable human keratinocytes
442E	Dermal sensitization in vitro	THP-1 monocytic leukemia cells
455	Estrogen receptor agonists and antagonists	ERα-HeLa-9903
456	Steroidogenesis	NCI-H295R cells
458	Transcriptional activation of stable receptor transfection human androgen	AR-EcoScreen cell line
460	Corrosion and severe eye irritation	MDCK CB997 cells
471	Bacterial reverse mutation	*Salmonella typhimurium* bacteria
473	Chromosomal aberrations in mammals	CHO, V79, CHL /IU, TK6 cells, primary lymphocytes
476	Cytotoxicity, genetic stability and gene mutations inmammals, like p53	CHO, CHL, V79, L5178Y and TK6 cells
487	Mammalian micro-nuclei	CHO, CHL, V79, L5178Y and TK6 cells
490	Cytotoxicity, mutations in mammalian genes such as that of timidin-kinase	L5178Y and TK6 cells
491	Assessment of severe eye damage	SIRC monolayer cells
492	Serious eye damage or irritation	Human keratinocytes or human cornea cells

^3^ Specific list of biological tests regulated by the OECD regulations that refer to the skin.

## Abbreviations

AD	Atopic Dermatitis
AhR	Aryl Hydrocarbon Receptor
BASS	Bioengineered Artificial Skin Substitutes
ECM	Extracellular Matrix
ER	Electrical Resistance
FBS	Fetal Bovine Serum
FDA	Food and Drug Administration
FLG	Filaggrin
GMP	Good Manufacturing Practices
GTP	Good Tissue Practices
HF	Human Fibroblasts
IL	Interleukins
IMT	Intima-Media Thickness
iPSC	Induced Pluripotent Stem Cells
ITG	Integrin
IVL	Involucrin
KG	Dipotassium Glycyrrhizinate
KRT	Keratin
MF	Murine Fibroblasts
MMP	Matrix Metalloproteinase
NK	Natural Killer Cell Conditioned Medium (NK-CdM)
NLC	Nanostructured Lipid Carrier
NR	Neutral Color
LDH	Lactate Dehydrogenase
LN	Laminin
LOR	Loricrin
NLC	Nanostructured Lipid Carrier
OECD	Organisation for Economic Co-operation and Development
OECD TG	Organisation for Economic Co-operation and Development Test Guideliness
PAMPA	Parallel Artificial Membrane Permeability Assay
ROS	Reactive Oxygen Species
SC	Stratum Corneum
TE	Tissue Engineering
TEWL	Transepidermal Water Loss
TIMP	Tissue Inhibitor Metallopeptidase
TSLP	Thymic Stromal Lymphopoeitin
TWF	Tritiated Water Flux
UV	Ultraviolet Radiation
VEGF	Vascular Endothelial Growth Factor
